# Expression and function of estrogen receptors and estrogen-related receptors in the brain and their association with Alzheimer’s disease

**DOI:** 10.3389/fendo.2023.1220150

**Published:** 2023-07-04

**Authors:** Kaoru Sato, Ken-ichi Takayama, Satoshi Inoue

**Affiliations:** ^1^ Department of Systems Aging Science and Medicine, Tokyo Metropolitan Institute for Geriatrics and Gerontology (TMIG), Tokyo, Japan; ^2^ Integrated Research Initiative for Living Well with Dementia (IRIDE), TMIG, Tokyo, Japan

**Keywords:** estrogen, estrogen receptor, estrogen-related receptor, brain, Alzheimer’s disease

## Abstract

While estrogens are well known for their pivotal role in the female reproductive system, they also play a crucial function in regulating physiological processes associated with learning and memory in the brain. Moreover, they have neuroprotective effects in the pathogenesis of Alzheimer’s disease (AD). Importantly, AD has a higher incidence in older and postmenopausal women than in men, and estrogen treatment might reduce the risk of AD in these women. In general, estrogens bind to and activate estrogen receptors (ERs)-mediated transcriptional machineries, and also stimulate signal transduction through membrane ERs (mERs). Estrogen-related receptors (ERRs), which share homologous sequences with ERs but lack estrogen-binding capabilities, are widely and highly expressed in the human brain and have also been implicated in AD pathogenesis. In this review, we primarily provide a summary of ER and ERR expression patterns in the human brain. In addition, we summarize recent studies on their role in learning and memory. We then review and discuss research that has elucidated the functions and importance of ERs and ERRs in AD pathogenesis, including their role in Aβ clearance and the reduction of phosphorylated tau levels. Elucidation of the mechanisms underlying ER- and ERR-mediated transcriptional machineries and their functions in healthy and diseased brains would provide new perspectives for the diagnosis and treatment of AD. Furthermore, exploring the potential role of estrogens and their receptors, ERs, in AD will facilitate a better understanding of the sex differences observed in AD, and lead to novel sex-specific therapeutic approaches.

## 1 Introduction

Estrogens, a class of steroid hormones, are one of the major female sex hormones produced primarily in the ovaries and plays a crucial role in the development and maintenance of the female reproductive system and secondary sexual characteristics. Even in non-reproductive tissues and organs, estrogen exerts important effects on various physiological systems in the body, including bone health, cardiovascular health, and brain function in both female and male (1–6). Especially, its decline due to menopause or oophorectomy can lead to several health complications, such as metabolic syndrome, osteoporosis, sarcopenia, frailty, cardiovascular disease, and dementia (6–8).

**Figure 1:**
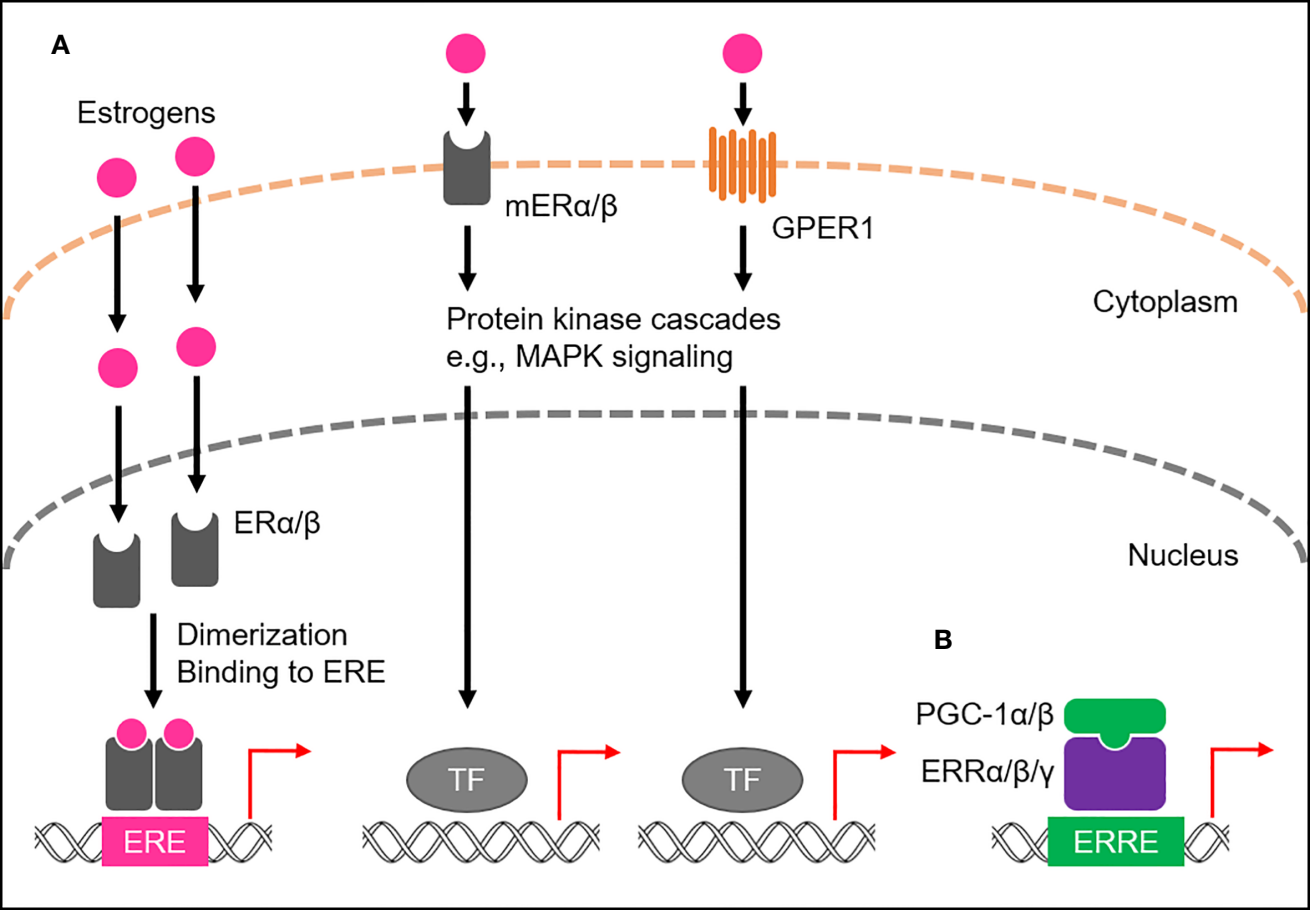
ER- and ERR-mediated transactional regulation. **(A)** Transactional regulation by ERs, namely ERα and ERβ, and membrane ERs (mERs). Endogenous estrogens bind to ERs or mERs. ERs bind to estrogen responsive elements (EREs) to activate transcription of target genes, while mERs (mERα/β and GPER1) mediate non-genomic effects of estrogens by stimulating activation of the protein kinase cascade, including MAPK signaling, which in turn activates nuclear transcription factors (TFs). **(B)** Transcriptional regulation by ERRs. ERRs predominantly localize to the nucleus and preferentially bind to estrogen-related receptor responsive elements (ERREs) to transcriptionally regulate the expression of target genes. PGC-1α/β act as co-activators to activate the transcriptional activity of ERRs.

Of the four major endogenous estrogens in women, estrone (E1), estradiol (E2), estriol (E3), and estetrol (E4), E2 is the most abundant throughout the reproductive lifespan, both in terms of its absolute serum concentration and its potent estrogenic activity (9, 10). Like all steroid hormones, estrogens can readily diffuse across the plasma membrane of cells (11). Inside a cell, they bind to and activate estrogen receptors (ERs), members of the NR3 subgroup of the nuclear receptor superfamily (12–14; **Figure 1A**). Once activated, ERs modulate the expression of multiple genes at the transcriptional level (15, 16). Humans possess the two primary types of ERs, namely ERα and ERβ, which activate gene transcription by binding to the genomic element known as the estrogen-response element (ERE), typically as a homo- or heterodimer with coactivators, such as steroid receptor coactivator-3 (SRC-3) and p300/CBP (17–19). Both ERα and ERβ are widely expressed in various human tissues, including the reproductive organs, breast tissue, bone, and brain, where they regulate the growth, development, and maintenance of these tissues (20). A subset of ERs associates with the plasma membrane, namely membrane-associated ERα (mERα) and ERβ (mERβ), and belongs to the membrane ERs (mERs) (21–24). These cell surface receptors rapidly activate estrogen signaling through intracellular signaling cascades (**Figure 1A**). In addition, another mER member, G protein-coupled estrogen receptor 1 (GPER1), also long known as G protein-coupled receptor 30 (GPR30), has been identified in various human tissues, including the reproductive organs, breast tissue, and brain (25–27; **Figure 1A**).

Estrogen-related receptors (ERRs), ERRα, ERRβ, and ERRγ, have been identified as nuclear receptors with substantial sequence similarity to ERs, thus belonging to the NR3 subgroup, but are orphan nuclear receptors because their endogenous ligands have long been unidentified and estrogens are also not their ligands (13, 14, 28; **Figure 1B**). Instead, their transcriptional activities are tightly regulated by the interactions of peroxisome proliferator-activated receptor-γ (PPARγ) coactivator 1-α (PGC-1α) and PGC-1β, suggesting the molecular function of both PGC-1α and PGC-1β as protein ligands for ERRs (29). ERRs are highly expressed in almost all human tissues, including skeletal muscle, fat, and brain (20, 30), where they play a role in regulating various physiological processes by transcriptionally activating multiple target genes by binding to the specific genomic element, called the estrogen-related response element (ERRE), as a monomer, homodimer or heterodimer (31–33).

This review focuses on the expression and functions of ERs and ERRs in the brain, mainly in humans. In addition, we discuss the implication of their roles in the pathogenesis of Alzheimer’s disease (AD).

## 2 ER function in the brain and association with AD

In this section, we summarize multiple neuronal functions of ERs and their association with AD.

### 2.1 Expression of ERs in the brain

The expression of both ER transcripts is widely observed in almost all cell types, namely neurons and glia, throughout the human brain, but with different expression patterns and levels (34, 35) The transcripts of *ESR1*, the gene encoding ERα, are predominantly expressed in the hypothalamus, amygdala, cerebellum, and cortex, while the transcripts of *ESR2*, encoding ERβ, are mainly expressed in the hippocampus and cortex, with lower expression levels than ERα (36). At the protein level, ERα immunopositive cells are first detected in the cortex at 9 weeks’ gestation (GW), especially in the proliferating zone and cortical plate, then gradually decrease during prenatal development, but increase again from birth to adulthood (37–40). ERα protein expression has also been demonstrated in the adult human hippocampus. In the human cortical tissue, ERβ initiates to be detected in the proliferating zones at 15 GW and in the cortical plate at 16-17 GW. Furthermore, ERβ protein expression persists into adulthood with a widespread distribution throughout cortical layers II-VI (37–40). Like ERα protein, ERβ has been detected in the human hippocampus from approximately 15 GW into adulthood, primarily in the pyramidal cells of Ammon’s horn and the dentate gyrus (40). Both ERs are expressed in neurons and glial cells in human cortical and hippocampal tissue during fetal development (40). Notably, higher expression of ERβ than ERα has been observed in the adult human cerebral cortex and hippocampus, suggesting that an important role of ERβ in the human brain (37–40). In the rat brain, besides the nuclear localization of ERs, mERα/β proteins are also found in complementary distributions in multiple regions, including the hippocampus and prefrontal cortex (41). Furthermore, GPER1 is widely distributed, with transcript and protein expression detected in nearly all regions of the adult human brain, particularly in the cerebral cortex, cerebellum, and basal ganglia (36, 42).

### 2.2 The physiological function of estrogens and ERs in the brain

Several factors, including aging and hormonal status, likely influence the expression of the ERs, ERα and ERβ, in the human brain. In the hippocampus of aged human brains, nuclear-localized ERα protein has been shown to increase in the dentate gyrus (DG) and CA3 region, while decreasing in the CA1 region (43, 44), suggesting changes in ERα-mediated transcriptional gene activation in the human brain during aging. In addition, treatment with a major estrogen, E2, increases nuclear-localized ERα in the human brain and maintains ERα-mediated transcription, compensating for hormonal loss during menopause (45). In contrast, GPER1 expression is unlikely to be affected by aging and surgical menopause (46).

The physiological effects of estrogen and ER expression on learning and memory have been better characterized in rodents, such as rats and mice, compared with in humans (47–51). In the rodent brain, estrogens act on the hippocampus, a complex brain structure that is primarily responsible for learning and forming new memories, where they acutely modulate the electrophysiological properties of hippocampal neurons (47, 49, 50). Through ERβ, E2 induces acute potentiation of excitatory postsynaptic currents (EPSCs) by selectively increasing glutamate release at synapses characterized by low initial release probability, while suppressing inhibitory neurotransmission in hippocampal CA1 neurons through ERα (52, 53). In addition, E2 causes a rapid increase in dendritic spine density in the CA1 region of the hippocampus (54) and can also rapidly enhance kainate-induced currents in hippocampal neurons even in the absence of ERs (55). Furthermore, even the membrane-impermeable estrogen, namely E2 conjugated to bovine serum albumin (E2-BSA), which cannot cross the plasma membranes of living cells, is capable of eliciting rapid estrogen signaling (56, 57). These observations suggest that, in addition to the nuclear ERα/β-mediated pathway, estrogens act through a rapid, membrane-initiated signaling pathway, likely mediated by mERα/β and/or GPER1, that activates multiple protein kinase cascades, including mitogen-activated protein kinase (MAPK) signaling, which in turn modulates synaptic plasticity and neuroprotection by stimulating the expression of multiple genes such as *brain-derived neurotrophic factor* (*Bdnf*), a master regulator of neuronal cell survival, synaptic plasticity, hippocampal function, and learning, in hippocampal neurons (58–61). Estrogens also undergo metabolic pathways such as sulfation and glucuronidation to form conjugated metabolites that inactivate E2 (62). The balance between these conjugated and unconjugated forms of estrogens in the brain may contribute to brain health and neuroprotection against the neurodegenerative process (63).

### 2.3 Estrogens and ERs associated with AD

AD is a progressive neurodegenerative disease that affects the brain and leads to cognitive, memory, and behavioral decline (64–67). The neuropathological hallmarks of AD are senile plaques and neurofibrillary tangles (NFTs) (68, 69). Senile plaques are extracellular structures composed predominantly of insoluble deposits of amyloid β peptide (Aβ) that are known to cause neuronal damage and neuronal cell death, while NFTs are aggregates of hyperphosphorylated tau protein within neurons that cause cell death and cognitive impairment in AD (65, 70–72). Aβ pathology likely precedes and accelerates tau pathology, which together trigger neurodegeneration and cognitive decline during AD development (65, 70, 73).

Notably, women have a higher lifetime risk of AD than men; the population of women with dementia is estimated to be approximately 1.69 times higher than the population of men with dementia worldwide, and they have approximately three times higher rates of disease progression with a broader range of cognitive symptoms (74–76). Importantly, despite some controversy, early estrogen replacement therapy (ERT), especially when given before menopause, has been shown to reduce the risk of AD in postmenopausal women (77). Estrogens have also been shown to have neuroprotective effects in the brain of rodent models of AD (78, 79). Moreover, increasing evidence suggests that ERs, including nuclear-localized ERα/β, mERα/β, and GPER1, play a role in AD pathogenesis (24, 45, 80, 81).

### 2.4 The role of ERs in AD pathogenesis

**Figure 2:**
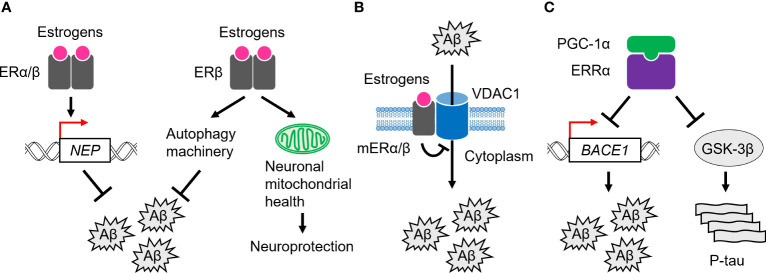
Role of ERs and ERRs in AD pathogenesis. **(A)** ERα and ERβ are involved in Aβ clearance through transcriptional activation of the *neprilysin* (*NEP*) gene. ERβ also activates the autophagy machinery to remove Aβ. In addition, it plays a neuroprotective role by regulating neuronal mitochondrial health. **(B)** mERα/β inhibit Aβ-induced neurotoxicity by inactivating a channel, VDAC1, at the plasma membrane. **(C)** ERRα, probably with PGC-1α, suppresses the expression of BACE1 and the kinase activity of GSK-3β, leading to Aβ clearance and tau phosphorylation (P-tau).

In the brain of AD patients, increased expression of the nuclear-localized ERα proteins has been observed in neurons of the basal forebrain, nucleus basalis of Meynert (NBM), medial mamillary nucleus (MMN), and hypothalamus, while decreased expression has been observed in hippocampal neurons (45, 80–86). Astrocytes are a subtype of glial cells in the brain and spinal cord (87), and increased numbers of nuclear ERα-positive astrocytes have been observed in the CA1 region of the hippocampus in AD patients (88). For Aβ clearance, ERα has been shown to upregulate the transcription of the Aβ degrading enzyme, *neprilysin* (*NEP*), in human cellular models of AD (89) (**Figure 2A**). Furthermore, ERα colocalizes with NFTs in the hippocampus of AD brains, and also physically interacts with tau protein, and this interaction is increased in AD brains (90). In addition, tau overexpression suppresses ERα transcriptional activity, suggesting that tau inhibits beneficial ERα signaling and neuroprotection through interaction with ERα in AD brains (90).

Nuclear ERβ proteins were significantly increased in the human NBM and hippocampal neurons in AD patients, whereas less ERβ was expressed in frontal cortex neurons (80, 82, 91). In SH-SY5Y and Swedish mutant (K670N/M671L) amyloid-β precursor protein (APP)-expressing HEK293 cells, which are human cell models of AD, ERβ promotes Aβ degradation by interacting with autophagy related 7 (ATG7) and further enhancing the autophagy machinery (92; **Figure 2A**). Like ERα, ERβ also upregulates the *NEP* transcription to promote Aβ clearance in cellular models (89; **Figure 2A**). These observations suggest that ERβ plays a beneficial role in Aβ clearance in AD. Notably, downregulation of several oxidative phosphorylation (OXPHOS)-related proteins, including ATP synthase subunits and cytochromes, has been demonstrated in the temporal cortex of women with AD and cerebrovascular disease (93).  Furthermore, in women, ERβ is associated with the mitochondria in the frontal cortex, and mitochondrial-localized ERβ proteins are decreased in the frontal cortex of women with AD (91). Given that ERβ loss impairs the mitochondrial membrane potential and function, it plays a neuroprotective role in modulating neuronal mitochondrial health modulating mitochondrial health (94–96; **Figure 2A**).

In the human cortex and hippocampus, mERα associates with voltage-dependent anion-selective channel 1 (VDAC1) at the plasma membrane to inactivate VDAC1 through phosphorylation, which in turn inhibits Aβ cellular entry and Aβ-induced cell death (97–100; **Figure 2B**). In support of this, activation of mERα/β alone inhibits Aβ-evoked neurotoxicity, oxidative stress, and apoptosis in the mouse primary neurons (101). Indeed, a reduced association of mERα with VDAC1 has been observed in the human cortex of AD brains (100, 102), suggesting the anti-AD capacity of mERα/β.

The neuroprotective contribution of GPER1 in AD has been highlighted in the rodent AD models (103). GPER1 inhibits Aβ-induced oxidative stress and neuronal cell death in rat neuronal cells (104). Moreover, GPER1 has been observed to stimulate extracellular signal-regulated kinase (ERK) signaling in rat hippocampal neurons, leading to activation of synaptic NMDA receptors and trafficking of AMPA receptors into hippocampal synapses, which in turn causes a persistent increase in synaptic efficacy, suggesting a role for GPER1 in modulating neuronal plasticity in neurodegenerative diseases, including AD (105).

## 3 ERR function in the brain and association with AD

We have reviewed the association between ERRs and AD pathogenesis.

### 3.1 ERR expression and function in the brain

In humans, the ERRα transcripts, *ESRRA*, and proteins are widely and highly expressed in almost all regions of the brain, including the hippocampus, cerebral cortex, and cerebellum (36). The ERRγ transcripts, *ESRRG*, are also detected throughout the human brain, but protein expression was detected at low levels in the cerebral cortex and cerebellum, and undetectable in the hippocampus. Notably, these two ERR proteins localize to neuronal cells, but not to glial cells, within their expressed regions in the human brain. In contrast, the ERRβ transcript, *ESRRB*, and protein expression are observed at no or very low levels in the human brain (36).

The roles of ERRα and ERRγ in memory and learning have been more extensively studied in rodents compared with in humans (59, 106, 107). The RNA expression pattern of all three ERRs, *Esrra*, *Esrrb*, and *Esrrg*, in the rodent brain is similar to that in the human brain, and the ERRα and ERRγ proteins are abundantly expressed throughout the mouse brain, including the cortex and hippocampus (36, 106, 108, 109), wheras ERRβ is primarily expressed in the developing mouse brain (110). Loss of neuronal ERRγ in the cortex and hippocampus impairs spatial learning and memory in mice (107). Long-term potentiation (LTP) is further impaired in ERRγ-deficient hippocampal neurons, which are rescued by supplementation of the mitochondrial substrate for ATP generation, pyruvate, suggesting a role for ERRγ in regulating neuronal cell metabolism (107). In contrast, the cognitive abilities of ERRα knockout mice were comparable to those of wild-type littermates (106). Meanwhile, endurance exercise increases hippocampal *fibronectin type III domain containing 5* (*Fndc5*) gene expression in mice through an ERRα/PGC-1α transcriptional complex, which in turn stimulates *Bdnf* gene expression, suggesting that ERRα may act as a mediator of exercise-induced beneficial effects that enhance cognitive function, including learning and memory.

### 3.2 Role of ERRs in AD

Accumulating evidence suggests a role for the ERR-mediated transcriptional machinery in AD pathogenesis. In APP-expressing HEK293 cells, ERRα inhibits Aβ production (111; **Figure 2C**). ERRα also downregulates the protein expression level of β-site amyloid precursor protein cleaving enzyme 1 (BACE1), the major β-secretase for Aβ production in neurons (112). However, it has not been demonstrated whether *BACE1* gene expression is directly regulated by ERRα at the transcriptional level. Furthermore, ERRα attenuates phosphorylated tau levels with a concomitant reduction in the phosphorylation of glycogen synthase kinase 3β (GSK-3β), an active form of the potent kinase for tau hyperphosphorylation (111; **Figure 2C**). In the APP/PS1 mice, a mouse model of AD that harbors human transgenes for both APP with the Swedish mutation and presenilin-1 (PSEN1) with the L166P mutation, ERRα RNA and protein expression levels are reduced in the cortex and hippocampus (111). In addition, PGC-1α RNA and protein expression levels are decreased in the AD brains with disease severity (113). Hippocampal PGC-1α protein content is inversely correlated with total Aβ content (113). Although the role of ERRγ in the pathogenesis of AD remains largely unexplored, despite its abundant expression in the human brain, these observations suggest that the ERR/PGC-1α transcriptional complex plays an important role in suppressing both Aβ and tau pathology throughout the progression of AD.

## 4 Conclusion

Strong evidences suggest that ERs and ERRs play important roles in human brain function, including learning and memory, as well as in the pathogenesis of AD, including the protection against Aβ-induced neurotoxicity and reduction of tau phosphorylation. Overall, however, the molecular mechanisms underlying the neuroprotective effects of ERs and ERRs in AD remain to be elucidated. Further studies are required to fully understand their roles in brain function and AD pathogenesis, which may lead to the development of novel therapeutic targets for the treatment of AD. In addition, exploring the potential roles of sex hormones, including estrogens, and their receptors in AD will help to better understand the sex differences observed in AD, and further lead to new sex-specific therapeutic approaches.

## Author contributions

All authors listed have made a substantial, direct, and intellectual contribution to the work and approved it for publication.
